# Sequence Properties of an Intramolecular Interaction that Inhibits p53 DNA Binding

**DOI:** 10.3390/biom12111558

**Published:** 2022-10-25

**Authors:** Emily Gregory, Gary W. Daughdrill

**Affiliations:** Department of Cell Biology, Microbiology, and Molecular Biology, University of South Florida, Tampa, FL 33620, USA

**Keywords:** tumor suppressor p53, intrinsically disordered proteins, intramolecular interaction, salt-dependent binding affinity, counterion condensation theory, DNA binding, fluorescence anisotropy, van’t Hoff, hydrodynamic radius

## Abstract

An intramolecular interaction between the p53 transactivation and DNA binding domains inhibits DNA binding. To study this autoinhibition, we used a fragment of p53, referred to as ND WT, containing the N-terminal transactivation domains (TAD1 and TAD2), a proline rich region (PRR), and the DNA binding domain (DBD). We mutated acidic, nonpolar, and aromatic amino acids in TAD2 to disrupt the interaction with DBD and measured the effects on DNA binding affinity at different ionic strengths using fluorescence anisotropy. We observed a large increase in DNA binding affinity for the mutants consistent with reduced autoinhibition. The ΔΔG between DBD and ND WT for binding a consensus DNA sequence is −3.0 kcal/mol at physiological ionic strength. ΔΔG increased to −1.03 kcal/mol when acidic residues in TAD2 were changed to alanine (ND DE) and to −1.13 kcal/mol when all the nonpolar residues, including W53/F54, were changed to alanine (ND NP). These results indicate there is some cooperation between acidic, nonpolar, and aromatic residues from TAD2 to inhibit DNA binding. The dependence of DNA binding affinity on ionic strength was used to predict excess counterion release for binding both consensus and scrambled DNA sequences, which was smaller for ND WT and ND NP with consensus DNA and smaller for scrambled DNA overall. Using size exclusion chromatography, we show that the ND mutants have similar Stokes radii to ND WT suggesting the mutants disrupt autoinhibition without changing the global structure.

## 1. Introduction

In response to cellular stress, the p53 tumor suppressor binds promoter response element DNA, activating transcription by recruiting the general transcription machinery [1,2,3]. Transcribed genes control cell fate decisions including cell cycle arrest, senescence, and apoptosis [4]. It is the most frequently mutated gene found in cancer, and mutations that interfere with DNA binding are found in a large subset of solid tumors [5,6,7]. p53 DNA binding and transcriptional activation is regulated by posttranslational modification, accumulation level, and association with other cellular factors [3,6,7,8,9,10]. p53 binds a 20 base pair DNA sequence consisting of two inverted repeats with the degenerate consensus sequence RRRCWWGYYY, where R is A/G, W is A/T, and Y is C/T [11,12]. p53 binds DNA as a homodimer to one 10 base pair repeat. The binding of a dimer to one repeat recruits a second dimer to the second repeat in a highly cooperative manner [13,14,15] and this homotetramer is the functional form of p53 [16,17]. Binding affinity of p53 to promoter DNA correlates with transactivation of genes, with dissociation constants (K_D_) ranging over three orders of magnitude, from low nanomolar to low micromolar, and binding affinity is higher for promoters that control cell cycle arrest and lower for promoters that control apoptosis [12,17,18,19,20,21]. Like most DNA-binding proteins, p53 binds both specific and nonspecific DNA [8,13].

**Figure 1:**
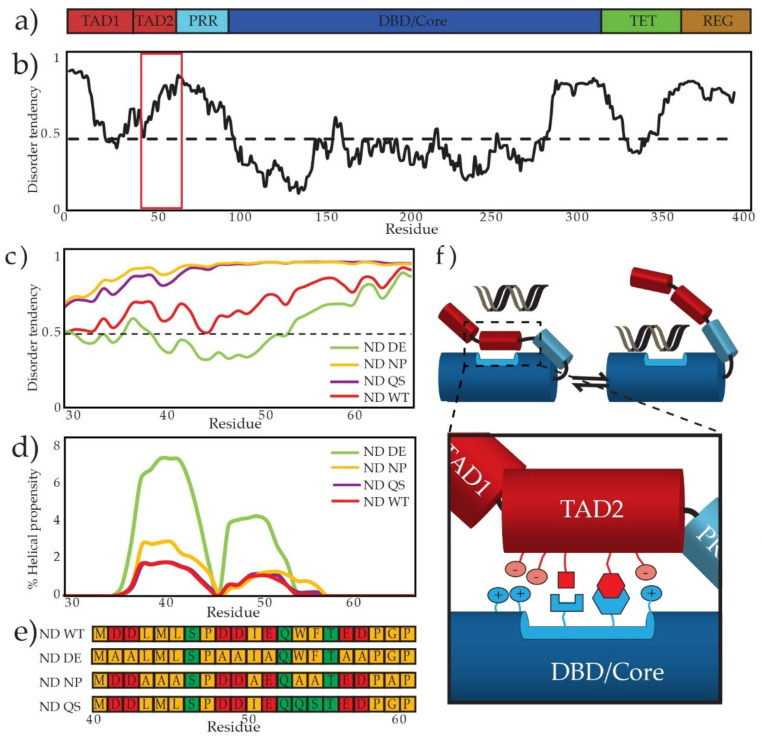
p53’s disordered TAD2 interacts with DBD. (**a**) A domain map shows p53’s domains. (**b**) IUPRED plot of full length p53 WT predicts regions of disorder based on sequence. The red box defines the region containing TAD2. (**c**) Inset from red box in (**b**) of IUPRED plot of a region containing TAD2 compares the disorder prediction of the wild type TAD2 and three mutants, where residues above the 0.5 line are predicted to be disordered. (**d**) Agadir prediction of helical propensity of the TAD2 region using wild type TAD2 and three mutants. (**e**) TAD2 sequences of the WT and mutants used in this study; red boxes indicate negatively charged residues, green boxes indicate polar residues, and gold boxes indicate nonpolar residues. (**f**) TAD2 interacts with DBD, inhibiting DNA binding by a combination a charge-based and specific interactions.

p53’s DNA binding affinity is regulated by an autoinhibitory intramolecular interaction between the disordered N-terminal transactivation domain (TAD) and the ordered DNA binding domain (DBD), resulting in a lowered DNA binding affinity and an increase in specificity for target DNA [22,23]. As shown in Figure 1a,b, the domain structure of p53 is defined as a TAD that is further divided into TAD1 (1–39) and TAD2 (40–60), followed by a proline rich region (PRR, residues 61–93), a DNA-binding or Core domain (94–292), a linker (293–322), a tetramerization domain (TET, residues 323–355), and regulatory domain (REG, residues 356–393).

The p53 intramolecular interaction primarily involves TAD2 and PRR [22] with a small contribution from TAD1 [24]. TAD2 is acidic and phosphorylation of TAD2 modulates DNA binding affinity [24]; additionally, the intramolecular interaction is neutralized at high salt concentrations [23] which suggests a strong electrostatic component. However, NMR data implicate several of TAD2’s noncharged residues in the interaction, pointing to a more complicated mechanism than merely the attraction of a negatively charged TAD with a positively charged DBD [22]. It is notable that the TAD2-DBD interaction does not confer a stable secondary structure to TAD2; it remains disordered even when bound to DBD [22]. The persistent disorder of the bound state is common in intrinsically disordered regions (IDRs) and is thought to decrease the entropic penalty of association [25,26]. A dynamic bound state is also observed in other IDRs that autoinhibit DNA binding like Ets-1 and HMGB1 [27,28]. The interaction between TAD2 and DBD is too weak *in trans* to measure by ITC. NMR data suggests a K_D_ in the micromolar to millimolar range [22]. Despite this weak interaction, when TAD2 is tethered to DBD by PRR, the free energy of binding to a consensus DNA sequence is decreased by 3 kcal/mol. The tethering of TAD to the DBD increases the effective concentration as is seen in other examples of disordered regulatory regions, where *in trans* binding affinities range from the low micromolar to the low millimolar and yet have large effects on DNA binding affinity [29,30]. There is some evidence from our group and others that TAD2 from one subunit of the dimer contacts the DBD from the other subunit [22,24] and the intramolecular interaction we observed for a p53 monomer may become intermolecular in the dimer.

IDRs are enriched in transcription factors [31,32,33], and their contribution to promoter selection is increasingly recognized [33,34,35,36]. The mechanism IDRs use to inhibit DNA often appears to rely on negatively charged residues in the IDR screening the DNA binding pocket. This can result from a disordered acidic domain interacting with a positively charged DNA-binding domain, as observed for the FOXO transcription factors [36], RFX1 [37], the HMG box family member UBF [38], HMGB1 [27], RBBP1 [30], the Sox transcription factors [39], and p53 [22,23]. IDRs can also inhibit DNA binding when phosphorylated, as seen for B-Myb [40] while Ets-1 uses a combination of phosphorylated serines and aromatic residues to tune inhibition [28,41].

Because the intramolecular interaction between TAD2 and DBD is weak *in trans*, we assess the interaction *in cis* using DNA binding. We introduce mutations to TAD2 that are predicted to weaken the intramolecular interaction and lead to increased DNA binding affinity. Because TAD2 lacks secondary structure in its apo and DBD-bound states, we used predictive tools to assess our designed mutants. An IUPRED plot predicts changes to disorder of TAD2 (Figure 1c) [42] and the Agadir plot of helical propensity (Figure 1d) [43] shows predicted changes for the mutants. This study uses DBD (94–312), a fragment containing the N-terminus and DBD (ND; 1–312), and mutants of ND (shown in Figure 1e) with substitutions where 7 acidic residues were changed to alanine (ND DE), where 7 nonpolar residues were changed to alanine (ND NP), and where W53/F54 were changed to QS (ND QS). Figure 1f shows a model of the TAD2-DBD interaction with an emphasis on charged and nonpolar interactions. Because the interaction is dynamic, there not a single structure that corresponds to the autoinhibited state. However, we assume charge-charge and nonpolar-nonpolar interactions occur even if there is multivalency [44]. Using high and low affinity DNA sequences, we compare the ability of the TAD2 mutants to inhibit DNA binding across a range of ionic strength (IS) with the expectation that electrostatic features of the TAD2-DBD interaction will be more sensitive to changes in salt concentration than nonelectrostatic features.

## 2. Materials and Methods

### 2.1. Protein Expression

Synthetic cDNA fragments of p53 (Genscript, Piscataway, NY, USA) were ligated into the pGEX-6P-1 plasmid (Sigma-Aldrich, Burlington, MA, USA) using BamHI and EcoRI restriction sites. cDNA for the ND DE and ND NP mutants were synthesized and the ND QS mutant was generated by site-directed mutagenesis starting with ND WT using Agilent’s Quikchange II protocol and kit (Santa Clara, CA, USA). All p53 fragments contain four stabilizing mutations in DBD: M133A, V203A, N239Y, and N268D [45]. Plasmids were transformed and expressed in BL21 (DE3) *E*. coli using minimal media at 37 °C to an O.D. of 0.5 at which point the media was supplemented with 20 μM ZnCl_2_, allowed to cool to 15 °C, and induced with 1 mM IPTG for 20 h. Cells were centrifuged at 7168 rcf for 5 min and frozen at −80 °C. To purify protein, one liter of pelleted cells was resuspended in 25 mL lysis buffer containing 50 mM Tris (pH 7.4), 500 mM NaCl, 2 mM DTT, 0.02% NaN_3_ and a fresh tablet of Pierce EDTA-free protease inhibitor (Thermo Fisher, Waltham, MA, USA). Cells were lysed via French press at approximately 1000 psi and centrifuged at 38,000 rcf for one hour. The supernatant was passed through a GST Fast-Flow Sepharose column (Cytiva, Marlboro, MA, USA) and eluted with 10 mM reduced glutathione. The eluted fractions containing the GST-tagged ND fragments were pooled and incubated with a 1:100 ratio of the HRV3C protease overnight at 4 °C to cleave the GST tag. The cleaved GST tag was removed by passing the mixture over another GST column. Following separation of p53 and the GST tag, fragments containing the TAD were dialyzed into a low-salt buffer and passed through a Q Sepharose High Performance anion exchange column (Cytiva), eluted in buffer containing 20 mM Tris at a pH of 7–8 depending on isoelectric point of the protein, 0–1 M NaCl, 2 mM DTT, and 0.02% NaN_3_. All fragments were analyzed using polyacrylamide gel electrophoresis and protein samples were pooled and concentrated 25–50 μM and loaded on a 16/600 mm Superdex 75 column (Cytiva) in a buffer composed of 50 mM NaH_2_PO_4_ (pH 7), 300 mM NaCl, 1 mM DTT, and 0.02% NaN_3_. Protein purity was evaluated via SDS-PAGE and concentration assessed using a Nanodrop 1000 Spectrometer (Thermo Fisher).

### 2.2. Preparation of DNA

HPLC-purified, 6-Carboxyfluorescein (6-FAM) tagged DNA was obtained from IDTDNA (Coralville, IA, USA) as single strands. Double-stranded DNA was annealed by boiling at 95 °C for 10 minutes and allowing to cool to room temperature. The sequences used are as follows: consensus 5′ AGACATGCCTAGACATGCCT and scrambled 5′ TGCCGATCAAAACCGATTCG. Annealing was confirmed using nondenaturing gel electrophoresis.

### 2.3. Fluorescence Anisotropy

Purified samples of DBD, ND WT, ND DE, ND NP, and ND QS were concentrated to 20–200 μM depending on the IS of the buffer and co-dialyzed with DNA twice against a buffer containing 10 mM NaH_2_PO_4_ (pH 7.4), 30–200 mM NaCl, 5 mM DTT, 0.02% NaN_3_, and 0.01% Triton-X 100 for a total dilution factor of 1 × 10^6^. 10 nM labeled DNA was aliquoted into Corning™ COSTAR 96-Well Solid Black Polystyrene Microplates (Thermo Fisher) and protein samples were added at increasing concentrations from 1 nM to saturation at 20–100 μM for a total volume of 100 μL. Fluorescence was measured using a Synergy H1 microplate reader from Biotek (Winooski, VT, USA) at 25 °C, and at 1.5° increments from 21–37 °C for van’t Hoff analysis. Excitation and emission wavelengths were 485 nm and 528 nm, respectively, with a sample height of 7 cm, gain of 50, and shake and delay steps of 30 s and 20 s, respectively.

Binding affinities were estimated using a cooperative binding model for p53’s interaction with consensus DNA as described previously [13] where p53 is evaluated as a dimer:

(1) $$\Delta A=\frac{{\left[p\right]}^{2}}{K_{D}+{\left[p\right]}^{2}}$$

Where ΔA is the normalized anisotropy change, [p] is p53 dimer concentration. Binding affinity to scrambled DNA was calculated using a one-to-one binding model [46]:

(2) $$\Delta A=\frac{\left[p\right]+\left[\mathrm{DNA}\right]+K_{D}-\sqrt{{\left(\left[p\right]+\left[\mathrm{DNA}\right]+K_{D}\right)}^{2}-4\left[p\right]\left[\mathrm{DNA}\right]}}{2\left[\mathrm{DNA}\right]}$$

The Hill coefficient was evaluated using the following equation [46]:

(3) $$\Delta A=\frac{{\left[p\right]}^{h}/K_{D}{}^{h}}{1+{\left[p\right]}^{h}/K_{D}{}^{h}}$$

where *h* is the Hill coefficient, indicating the cooperativity of the binding event where 1 is a noncooperative event and greater than 1 is a cooperative event.

Enthalpy and entropy estimates were calculated from van’t Hoff plots. These were generated by measuring anisotropy at physiological IS across a range of temperatures as previously described [47].

### 2.4. Estimating Counterion Release

The counterion condensation theory developed by Record and colleagues expands on the polyelectrolyte theory [48] to estimate ionic contacts and excess ion release for protein-nucleic acid binding [49] using the following relationship:

log(K_A_) = log(K^′^_A_) − N*log[Salt] (4)

Where K_A_ is the association constant, K^′^_A_ is the nonelectrostatic component of binding, and N*log[Salt] is the electrostatic component of binding. *N* is the slope of a double log plot of K_A_ versus [Salt]. In this theory the electrostatic component of binding refers to the positive entropy associated with ion release [49,50]. It is unclear if this approach can quantitatively discriminate the salt-dependent entropic component of binding from other components, but we think it provides a useful qualitative segregation of components of binding affinity [51,52]. Because of this we refer to it these as the salt-dependent and salt-independent components of binding rather than as the electrostatic and nonelectrostatic components. The salt-independent component is inferred from the y-intercept of a log(K_A_) vs. log[Salt]. The slope of this plot, *N*, is further defined as:

*N* = ZΨ + β (5)

where Z is the number of protein-DNA backbone contacts made, Ψ is the fractional number of ions bound by phosphate, 0.7 for short oligonucleotides [53], and β is the number of excess ions released from protein. Our study utilizes only NaCl as the salt. Studies have found that variation of the monovalent cation, which is condensed around and ultimately released from DNA, is unimportant in evaluating ion release [50,54] although introduction of a divalent cation can have complicated effects on apparent ion release [55]. Variation of the anion may affect apparent ion release; however, the change in apparent ion release based on anion identity may reflect on the size of the anion or its relative attraction to water versus the protein side chains and thus varying the anion is not predicted to reveal additional information about the protein’s DNA binding interface [50,56,57]. 

A reevaluation of the theory by Manning and colleagues resulted in the following relationship [58]:

log(K_A_) = log(K_0_) + log V + 0.513Z − 0.434 − Z*log[Salt] (6)

where K_A_ is the association constant, K_0_ is the salt-independent component of binding, V is the reaction volume, and Z represents the number of charged molecules associated with the binding event, which is interchangeable with *N* from Equation (2).

Both these approaches use the section of a double log plot where log(K_A_) versus log[Salt] becomes linear, a range that is uniquely determined for a given protein. In this case, while fluorescence anisotropy was conducted on DBD and ND WT over an IS range of 15–225 mM, Supplementary Table S1 , the double log plot is linear in the 125–225 mM range. Thus, fluorescence anisotropy was only conducted on ND mutants in the 85–225 mM range and these were evaluated using the counterion condensation theory from 125–225 mM IS.

### 2.5. Size Exclusion Chromatography

Stokes radii (R_H_) of the p53 fragments were determined using size exclusion chromatography (SEC). The Cytiva Gel Filtration Calibration Kit LMW was used to generate a calibration curve in a buffer of 50 mM NaH_2_PO_4_, pH 7.4, 300 mM NaCl, 0.02% NaN_3_ using a HiLoad 16/600 mm Superdex 75 column (Cytiva, Marlboro, MA, USA) at 4 °C. A high ionic strength buffer was used to reduce binding to the sephadex beads and decrease line broadening. The elution volume of each protein was taken as the average of three injections, each of which contained 0.6–0.8 mg/mL of protein. The peak elution volume is used to find the partition coefficient, K_av_:

K_av_ = (V_t_ − V_o_)/(V_c_ − V_o_) (7)

Where V_c_ is the total column volume, V_o_ is the void volume, and V_t_ is the elution volume. A plot of log(K_av_) versus the known R_H_ of calibration kit standards generates a trendline from which R_H_ of an unknown protein can be estimated [59,60]. Error of R_H_ values is determined by the average of three runs where the resolution of the elution volume is 0.02 mL. We acknowledge previous work by Langridge and Whitten showing that the hydrodynamic radius of TAD increases with decreasing temperature [61]. 

## 3. Results

### 3.1. Salt Dependence of p53 DBD Binding DNA

**Figure 2:**
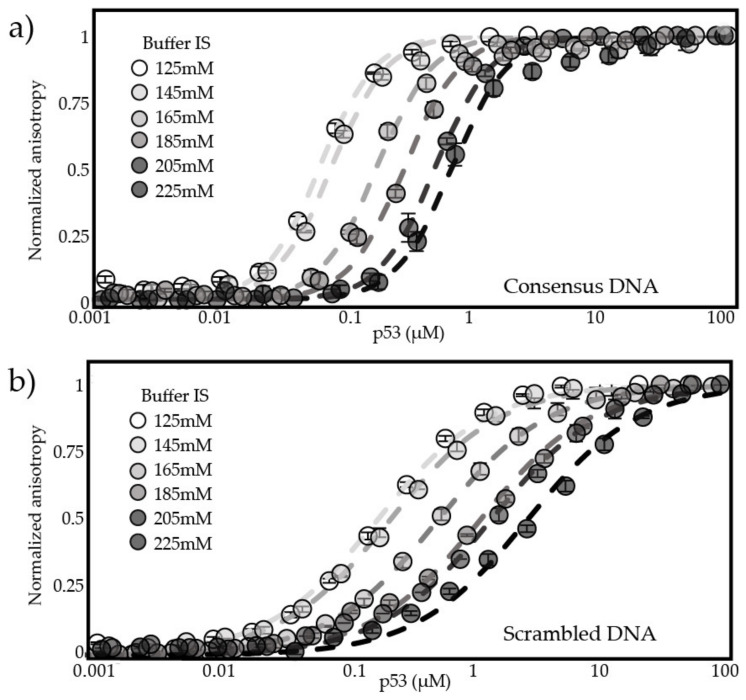
DBD binds DNA across IS. Fluorescence anisotropy plots show the change in signal from a fluorescently tagged DNA fragment as protein is added: an increase in the concentration of p53 needed to achieve saturation when DNA concentration is kept stable as buffer salt concentration increases. (**a**) fluorescence anisotropy plots of DBD bound to consensus DNA at 125–225 mM IS; (**b**) fluorescence anisotropy plots of DBD bound to scrambled DNA at 125–225 mM IS.

We conducted binding experiments using fluorescence anisotropy in buffers with IS ranging from 15–225 mM. We used two DNA sequences. One is a high affinity sequence taken from a consensus promoter sequence [62], which we refer to as consensus DNA. The other is a scrambled version of this sequence that maintains the same GC content and is used as a representative of nontarget DNA. Figure 2 shows the normalized anisotropy values of fluorescently labeledDNA plotted as a function of DBD concentration. Dashed lines show the fit to a cooperative binding model in the case of consensus DNA (Figure 2a), and a single-site binding model was used to fit the data for scrambled DNA (Figure 2b). Both models assume p53 binds DNA as a dimer of dimers [13]. As salt concentration increases, binding affinity of DBD to DNA decreases. This is in accordance with observations of p53 specifically [13] and of DNA-binding proteins in general [63,64]. Hill coefficients are approximately 1.8 for DBD binding to consensus DNA and 1 for binding to scrambled DNA. This supports previous studies showing that p53 binds its target DNA in a cooperative manner and nontarget DNA in a noncooperative manner [13]. We observed the same trend in cooperativity when ND WT and the mutants bind to DNA, but K_D_ values are 5–200 times larger (Table S1 and Figure S1). At 125 mM IS the K_D_ for DBD binding consensus DNA was 0.9 ± 0.07 nM and at 225 mM IS K_D_ was 104.5 ± 5 nM. For binding to scrambled DNA, K_D_ ranges from 89.1 ± 5 nM to 1388 ± 44 nM over the same range of IS. These results are in the same range as previously observed binding affinities of DBD to DNA [22,65]. Similar trends are observed for ND WT, for which fluorescence anisotropy curves across a range of IS are shown in Figure S2. The K_D_ for ND WT binding to consensus DNA ranges from 43 ± 3.4 nM to 3861 ± 40 nM and binding to scrambled DNA ranges from 193 ± 8.2 nM to 3705 ± 230 nM. See Table S1 for full range of values. Error bars in Figure 2a,b represent the standard deviation of three measurements at each IS and the fitting errors presented in Table S2 are the standard error of estimate.

### 3.2. DBD, ND, and ND Mutants Binding to Consensus and Scrambled DNA at Physiological IS

**Figure 3:**
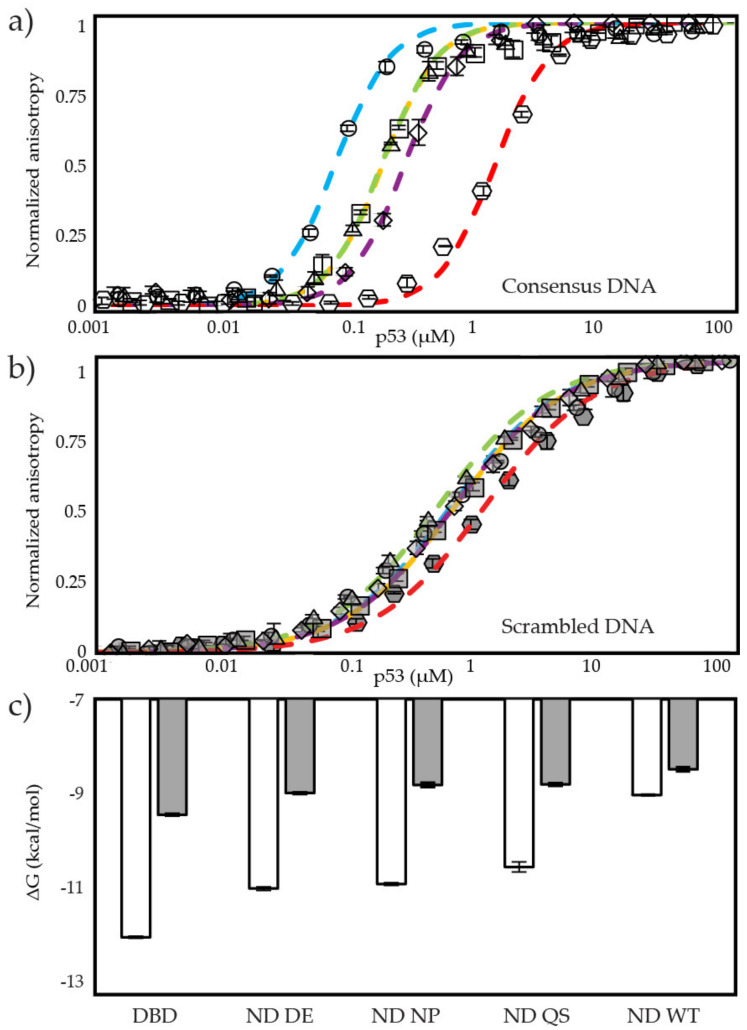
Binding of DBD and ND fragments to consensus and scrambled DNA at physiological IS (145 mM). (**a**) Fluorescence anisotropy plots of p53 constructs binding consensus DNA, where 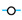 is DBD, 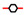 is ND WT,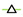 is ND DE, 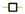 is ND NP, 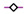 is ND QS, (**b**) p53 constructs binding scrambled DNA, where 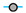 is DBD, 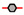 is ND WT, 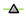 is ND DE, DNA, 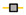 is ND NP, 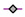 is ND QS, (**c**) ΔG of all fragments with consensus and scrambled DNA. Each data set represents three titrations.

To determine the contributions of charged and nonpolar interactions between TAD2 and DBD in the autoinhibition of DNA binding we designed three mutants where all aspartic and glutamic acid residues in TAD2 were changed to alanine (ND DE), where all the nonpolar residues from TAD2, including W53 and F54, were changed to alanine (ND NP), and where W53 and F54, were changed to glutamine and serine (ND QS) (See Figure 1e for sequences). The ND QS mutant is based on an early study of p53 in which this mutation inhibited transactivation and apoptosis by inhibiting interactions with multiple domains of CBP/p300 [66,67,68]. A decrease in the intramolecular interaction should lead to increased DNA binding affinity. Figure 3a shows the binding curves of fluorescence anisotropy experiments for DBD, ND WT, and the ND mutants at physiological IS (145 mM). The ND mutants have a binding affinity for consensus DNA that is closer to DBD than ND WT, indicating all the mutants disrupt the intramolecular interaction between TAD2 and DBD. ND DE and ND NP have similar binding affinities to one another for consensus and scrambled DNA, increasing the free energy of binding for consensus DNA relative to ND WT by −1.99 and −1.89 kcal/mol, respectively (Table 1). The ND QS mutant has DNA binding affinity between ND NP and ND WT and increases the free energy of consensus DNA binding by −1.49 kcal/mol relative to ND WT.

Figure 3b shows that binding affinities for the ND mutants with scrambled DNA are in a similar order as we observe for consensus DNA. ND DE increases the free energy of binding by −0.51 kcal/mol relative to ND WT; ND NP and ND QS both increase free energy of binding by −0.32 and −0.33 kcal/mol, respectively (Table 1 and Figure 3c). The ND fragments binding consensus DNA have a ΔΔG with DBD ranging from −1.03 kcal/mol to −3.02 kcal/mol. ND fragments binding scrambled DNA have a ΔΔG with DBD ranging from −0.46 kcal/mol to −1.04 kcal/mol. Similar to DBD, the ND fragments show cooperative binding to consensus DNA and noncooperative binding to scrambled DNA, as seen in Figure S1 where consensus DNA binding data points match a fit line with a Hill coefficient of 2 and scrambled DNA binding data points match a fit line with a Hill coefficient of 1. Thus, the intramolecular interaction does not affect cooperativity of DBD on target DNA. In summary, we find that introduction of mutations to TAD2 decreases the intramolecular interaction and increases DNA binding affinity. We find the ND DE mutant has the largest change in autoinhibition, followed by ND NP, and then ND QS.

### 3.3. Effects of IS on Binding Specificity of DBD, ND WT, and the ND Mutants

**Figure 4:**
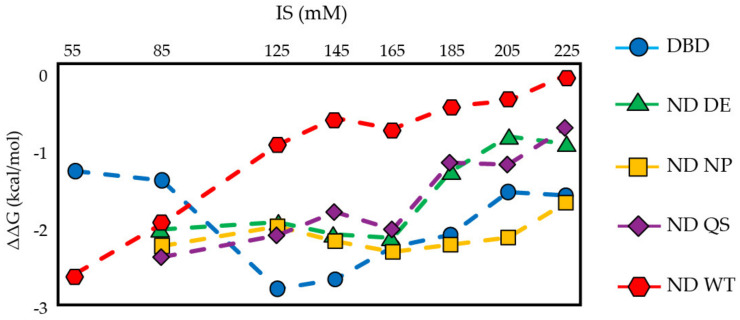
Binding specificity of DBD, ND WT, and ND mutants. For each p53 fragment, ΔΔG = ΔG_consensus_ − ΔG_scrambled_ at a given IS indicates binding specificity.

Binding specificity is commonly estimated as ΔG_specific_ − ΔG_nonspecific_ [69,70]. Figure 4 shows the ΔΔG values for DBD and ND WT at 55–225 mM IS, and the ND mutants at 85–225 mM IS. Below physiological ionic strength, ND WT has greater specificity than DBD for consensus DNA than scrambled DNA as evidenced by the larger negative ΔΔG; however, this trend reverses between 85–125 mM IS. Figure 4 also shows that at higher IS, ND NP has a similar binding specificity to DBD and the binding specificity for ND DE closer to ND WT. This is interesting because we expect the nonpolar interactions between TAD2 and DBD to be more specific than the charged interactions and our data shows that removing them increases DNA binding specificity while removing the charged interactions between TAD2 and DBD reduces specificity. We think ND DE has lower binding specificity because the strength of the hydrophobic effect between nonpolar residues in TAD2 and DBD becomes stronger at higher IS [71,72]. In contrast, ΔΔG for ND NP tracks with DBD at higher salt concentrations, indicating that the acidic residues in TAD2 are responsible for inhibiting binding to nonspecific DNA. We expect residues W53 and F54 in TAD2 to play a role in forming specific interactions with DBD but introduction of Q53/S54 reduces DNA binding specificity, suggesting the introduction of these amino acids, and not removal of W53/F54, is driving this effect. The ND WT fragment used in this study lacks the tetramerization domain and only enhances DNA binding specificity at low ionic strength even though it shows strong inhibition of DNA binding and maintains binding cooperativity for specific DNA up to 225 mM IS. As shown in Figure 4, the DBD can bind DNA specifically in the absence of TAD2 and the TET, and Figure 3c shows that ND WT inhibits binding to either consensus or scrambled DNA by a similar amount.

In our previous work we showed the intramolecular interaction between TAD2 and DBD in monomeric p53 became intermolecular when the tetramerization domain (TET) was present [22]. In a related study, Wright and colleagues showed that adding TAD2 to a p53 fragment containing the DBD and TET enhances DNA binding specificity by inhibiting binding to nonspecific DNA but has no effect on binding to specific DNA [23]. The binding studies by Wright and colleagues were conducted at an IS close to 165 mM using similar specific and nonspecific sequences to ours. Using full length p53 with and without TAD2, their K_D_ ratio for binding was 1 for specific DNA and 5.7 for nonspecific DNA. By comparison our K_D_ ratios for ND WT and DBD binding to specific and nonspecific DNA are 70 and 5.3, respectively. Taken together these data suggest that inhibition of DNA binding to both specific and nonspecific sequences is driven by the intramolecular interaction between TAD2 and DBD and specificity enhancement depends on this interaction becoming intermolecular when p53 is tetrameric. As mentioned, we think addition of the tetramerization domain reduces the hydrophobic effect between TAD2 and DBD and this could be happening due differences in the way TAD2 interacts with DBD when the intramolecular interaction becomes intermolecular.

### 3.4. Estimating Ion Release Using Counterion Condensation Theory

**Figure 5:**
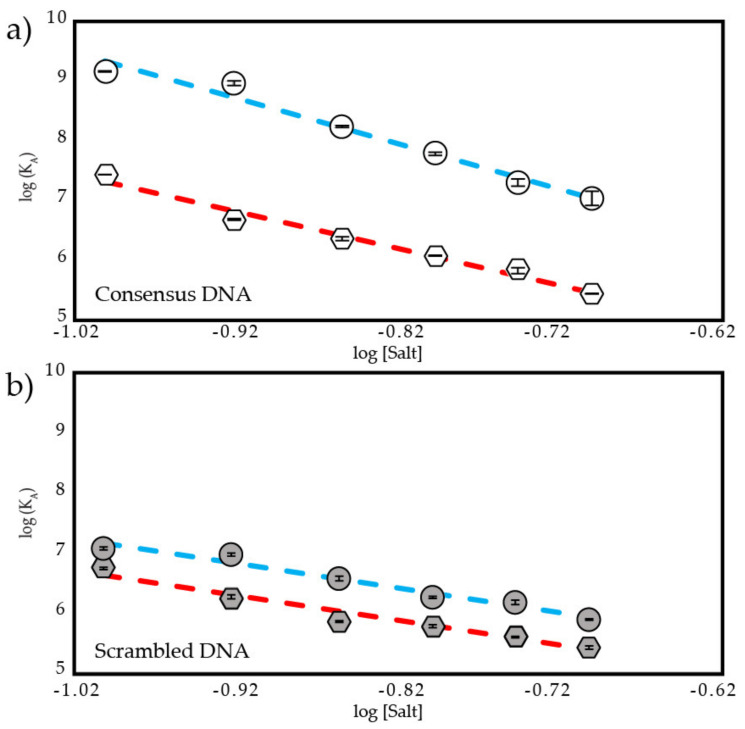
Salt-dependent binding affinity of DBD and ND WT. Plot of log (K_A_) vs. log [Salt] from 125–225 mM IS of (**a**) DBD and ND WT binding to consensus DNA where 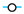 is DBD, 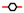 is ND WT, (**b**) DBD and ND WT binding to scrambled DNA where 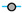 is DBD, 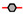 is ND WT. R^2^ values for all fit lines are between 0.96 and 0.99.

**Figure 6:**
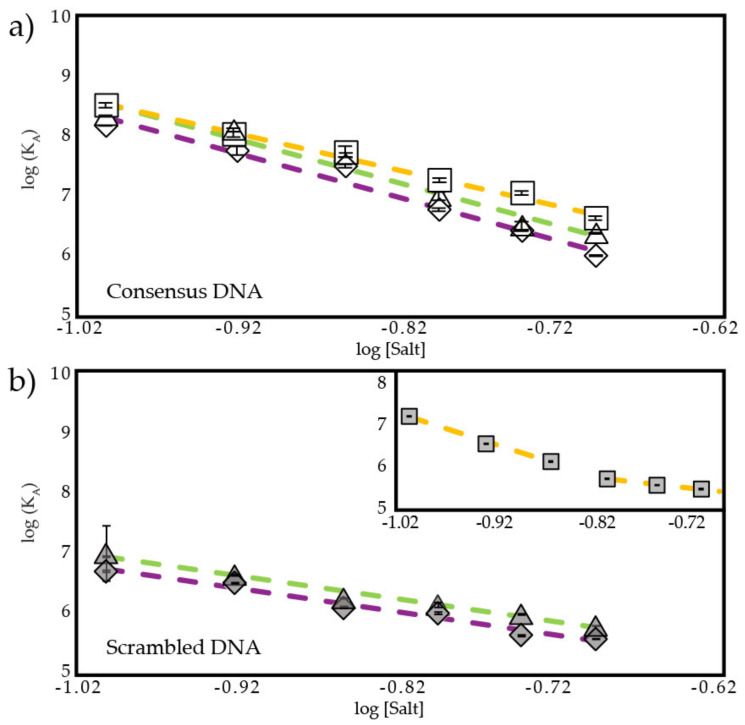
Salt-dependent binding affinity of ND mutants. Plot of log (K_A_) vs. log[Salt] from 125–225 mM IS of (**a**) ND mutants binding consensus DNA, where 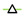 is ND DE, 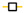 is ND NP, 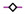 is ND QS (**b**) ND mutants binding scrambled DNA, where 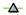 is ND DE, 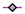 is ND QS. Inset shows ND NP binding scrambled DNA, 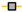. R^2^ values for all fit lines are between 0.96 and 0.99.

To assess the sensitivity of the TAD2-DBD interaction to IS, we conducted fluorescence anisotropy binding experiments on ND WT and the ND mutants from 125–225 mM IS. Figure 5 and Figure 6 show the linear region of log(K_A_) versus log[Salt] plots. Figure 5a shows that the binding of consensus DNA to DBD is tighter than to ND WT at every IS and that the presence of TAD2 in ND WT inhibits DNA binding at a level that corresponds to increasing IS by 70–80 mM for DBD. Binding of DBD and ND WT to scrambled DNA (Figure 5b) shows a similar trend in affinity where the inhibition of DNA binding by TAD2 corresponds to an increased IS of 40–60 mM for DBD. 

Counterion condensation theory proposes that ions are uniformly condensed on DNA at a concentration that is relatively independent of buffer conditions or the type of protein binding. When a positively charged protein binds DNA, a number of counterions equivalent (or fractionally equivalent) to the number of nonspecific ionic contacts made between the protein and DNA backbone are released into solution [48]. The oligolysine model developed by Record and colleagues as an extension of the counterion condensation theory predicts that the observed decrease in DNA binding affinity as salt concentration increases can be used to estimate the number of these nonspecific ionic contacts [49,73]. In Equation (5), the slope (*N*) of the double log plots in Figure 5 and Figure 6 is proportional to the fractional number of counterions released from the DNA backbone (Ψ), approximately 0.7 per phosphate contact for short oligonucleotides [53], and any excess ions released from the protein (β). According to this theory, a smaller slope corresponds to release of fewer ions, whether they originate from backbone phosphates or from protein. As shown in Table 2, DBD has a larger slope than ND WT when binding consensus DNA, corresponding to greater predicted ion release. 

Crystallographic studies show five DNA backbone contacts made by DBD when bound to the p21 promoter [74,75]. We assume the same number of DNA backbone contacts are made by DBD to consensus DNA because our consensus sequence is similar to the p21 sequence. We also assume ND WT and ND mutants make the same number of contacts as DBD because TAD2 does not interact with DNA [22] or affect binding cooperativity according to the Hill plots in Figure S1. The difference in the slopes between DBD and ND WT when binding consensus DNA corresponds to a difference in the predicted release of excess ions when binding DNA (Table 2) where DBD is predicted to release 3.9 excess ions and ND WT is predicted to release 2.5 excess ions. This small difference in ion release corresponds to a difference in salt sensitivity where DBD experiences a 117-fold increase in K_D_ versus ND WT’s 86-fold increase in K_D_ over this range of IS. We also observe that inhibition of DNA binding is greater for ND WT as IS decreases, indicating a stronger intramolecular interaction at lower salt concentrations. A similar divergence of salt-dependent binding affinity was seen in a previous study of an autoinhibitory IDR-DBD interaction [27], in which the addition of an acidic domain lowered both DNA binding affinity and changed the slope of its double log plot. By contrast, ND WT binding to scrambled DNA has a slope similar to that of DBD (Table 2). We assume the same number of backbone contacts are made when p53 binds a nontarget sequence as is suggested by structures of low affinity p53-DNA complexes [75]. Assuming five backbone contacts, the slopes of ND WT and DBD when binding scrambled DNA correspond to predicted excess ion release of 0.7 and 0.6, respectively.

Figure 6a shows ND DE, ND NP, and ND QS bind consensus DNA more tightly than ND WT (also see Table S1). Slope values for ND DE and ND QS are close to DBD, while ND NP has a slope close to ND WT (Table 2). From these results we can make three conclusions: (1) ion release after removal of acidic residues (ND DE) is similar to ion release of DBD, (2) removal of several nonpolar residues in TAD2, including W53 and F54, (ND NP) has no effect on ion release relative to ND WT, and (3) introduction of Q53 and S54, not removal of W53 and F54, is responsible for changes in ion release of ND QS. The first two conclusions were expected and the third suggests the Q53/S54 mutant may do more than interfere with binding to CBP.

When binding scrambled DNA, the slopes are similar for DBD, ND WT, and the ND DE and ND QS mutants (Figure 6b). We predict that ND DE and ND QS release 0.5 and 0.4 excess ions, respectively, when binding scrambled DNA, similar to DBD and ND WT. ND NP does not have a single linear slope over the 125–225 mM range when binding scrambled DNA. Instead, it appears to have a linear portion at 125–165 mM IS with a slope of −6.91 and another linear portion at 185–225 mM IS with a slope of −2.35 as shown in the inset in Figure 6b. Slopes and estimated excess ion release from these two states are shown in Table 2 to be different from each other and from other p53 fragments. This suggests to us that ND NP binds scrambled DNA in multiple states.

**Figure 7:**
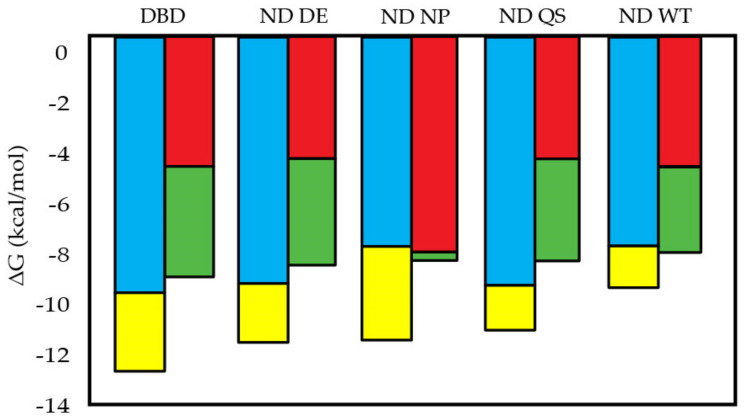
Salt-dependent and salt-independent components of Gibbs free energy at 145 mM IS from Record’s model. Free energy is apportioned into categories by assuming complete inhibition of salt-dependent components at 1M NaCl so the remainder of free energy is salt-independent. Slopes of double log plot slopes are used to estimate binding affinity at 1M NaCl, where 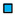 is the salt-dependent component and 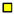 is the salt-independent component for consensus DNA and 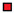 is the salt-dependent component and 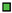 is the salt-independent component for scrambled DNA.

According to the oligolysine model, ΔG of binding can be separated into electrostatic and nonelectrostatic components, where the slopes of the plots in Figure 5 and Figure 6 multiplied by log[Salt] is the salt-dependent entropy due to ions being released into solution from the phosphate backbone [49,58]. As shown in Figure 7 and Table S3, the salt-dependent entropy is predicted to be the energetic driver of the p53 fragments binding to consensus DNA, ranging from 68–85% of the total energy. However, in an earlier binding study from our group at an IS of 85 mM using isothermal titration calorimetry we observed a large entropic penalty for DBD binding consensus DNA and a smaller penalty for ND WT and both had a large enthalpy change upon binding [22]. Van’t Hoff plots using temperature-dependent fluorescence anisotropy data also predict a large enthalpic component of binding (Figure S4 and Table S5) [76]. This suggests to us that for p53 the salt-dependent component of binding is not just made up of an entropic contribution from ion release. According to the Record model, the salt-dependent and independent contributions to binding free energy for DBD are predicted to be −9.30 kcal/mol and −2.77 kcal/mol, respectively, and for ND WT they are −7.55 kcal/mol and −1.50 kcal/mol, respectively. For all the fragments except ND NP, a smaller contribution for binding to scrambled DNA comes from the salt-dependent component. For DBD, the salt-dependent and independent components of binding to scrambled DNA are −5.14 kcal/mol and −4.32 kcal/mol, respectively, and for ND WT are −5.22 kcal/mol and −3.28 kcal/mol, respectively. An analysis of these components using Manning’s model, Equation (6), also predicts that salt-dependent entropy is a larger component of binding to consensus DNA than to scrambled DNA (Table S4 and Figure S3).

Salt-dependent ion release is one of several mechanisms that proteins use to achieve specificity in DNA binding. Studies have characterized systems in which the salt-dependent component of binding is higher for specific than nonspecific DNA binding [77], in which the salt-dependent component is similar for specific and nonspecific DNA binding [50,78], in which the salt-dependent component is lower for specific than for nonspecific DNA binding [57,79,80], in which the salt-dependent component is relatively low for both specific and nonspecific binding [47,81,82], and in which the salt-dependent component follows no clear trend between specific and nonspecific DNA binding [83,84]. It appears that our p53 fragments utilize salt-dependent components of the interaction for specific binding to a greater degree than the salt-independent components, and this trend is reversed for nonspecific DNA. Our mutants also follow this trend, with the exception of ND NP, which may switch between two modes depending on the IS.

In summary, using the salt-dependent component of binding, we find that predicted excess ion release upon protein-DNA binding is greater when our p53 fragments binding consensus DNA than scrambled DNA. Whereas excess ion release varies by fragment when binding consensus DNA, it is similar between all fragments when binding scrambled DNA excepting ND NP. This salt-dependent component comprises a variable amount of the free energy of binding for each fragment and generally comprises a greater amount of the free energy of binding for consensus DNA than scrambled DNA.

### 3.5. The Intramolecular Interaction Affects Stokes Radius and Apparent Molecular Weight

**Figure 8:**
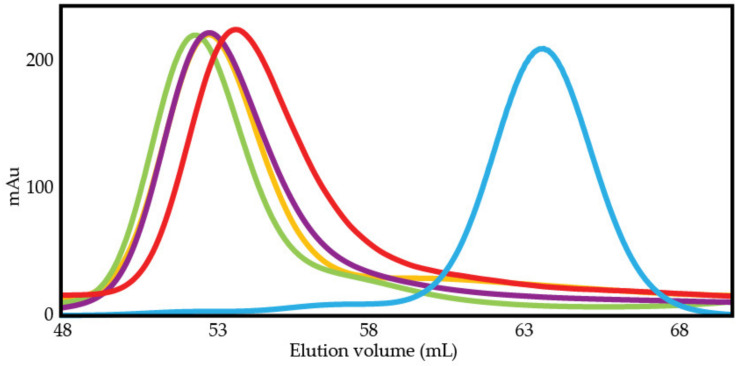
Size exclusion chromatography is used to compare p53 constructs. Elution profiles of p53 constructs where lower elution volume indicates a larger hydrodynamic radius: 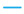 DBD, 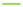 ND DE, 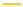 ND NP, 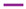 ND QS, 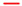 ND WT.

Using size exclusion chromatography (SEC) at high IS (410 mM), the elution volumes of p53 constructs were compared to elution volumes of known standards (see methods) to determine their Stokes radii and apparent molecular weights. As shown in Figure 8, ND mutant constructs elute at a lower volume than ND WT, which elutes at a lower volume than DBD. As shown in Table 3, we find the Stokes radius of DBD to be 2.74 ± 0.004 nm, in agreement with a previously published Stokes radius of the same DBD fragment using dynamic light scattering (2.74 nm) [85], whereas the radius of ND WT was found to be 3.55 ± 0.004 nm. The change in radius with the tethered TAD is relatively small given that p53 residues 1–93, including TAD1, TAD2, and PRR, has a Stokes radius of 3.5 nm at 5 °C [61]. ND WT appears to be more compact than predicted for 93 disordered residues attached to 219 ordered residues, but the ND WT is more expanded than predicted for a folded protein of the same number of residues (2.51 ± 0.59 nm) [86]. Estimating the hydrodynamic radius of a protein containing both ordered and disordered sections is an ongoing challenge [86,87]. Both DBD and ND WT have an apparent molecular weight greater than their actual molecular weight, as shown in Table 3. For DBD this is likely due to a disordered segment near the C-terminus from residues 292–312 (**PDB 4HJE**) [75]. ND WT and the ND mutants have apparent molecular weights almost twice as large as their actual molecular weights using this technique. 

We observe a small decrease in the elution volume of the ND mutants relative to ND WT, but it is larger than the resolution error of the volume measurement (+/−0.02 mL). Small changes in Stokes radii are evidence the mutants do not disrupt the global structure of ND, which was unexpected given the increase in DNA binding affinity of the mutants relative to ND WT. We suspect maintenance of the global structure is being driven by the PRR and will test this hypothesis in the future. We also conducted SEC on ND WT at 150 mM IS to test for changes in elution volume at low IS and compared this result to the elution volume at 410 mM IS. Shown in Figure S5, ND WT’s elution volume varies between these two conditions by <0.2 mL, a difference that corresponds to an approximately 0.03 nm difference in Stokes radius and less than 1 kDa difference in apparent molecular weight.

## 4. Discussion

We find that the intramolecular interaction between the TAD2 and DBD domains of p53 is disrupted by mutations targeting multiple types of interactions. Alanine substitutions of TAD2’s negatively charged residues, ND DE, increased consensus DNA free energy of binding by −1.99 kcal/mol relative to ND WT, suggesting that electrostatics play a large role in the intramolecular interaction and autoinhibition of DNA binding. Alanine substitutions of nonpolar residues, ND NP, increased DNA free energy of binding by −1.89 kcal/mol, suggesting a nonelectrostatic component. A targeted substitution of W53/F54 to Q53/S54, ND QS, chosen because of its established ability to disrupt other important TAD2 interactions [66,67,68], increases consensus DNA free energy of binding by −1.49 kcal/mol. The sum of the effects of the ND DE and ND NP mutants on the autoinhibition of DNA binding is 1 kcal/mol greater than the effect of ND WT. This indicates some cooperativity between the acidic, nonpolar, and aromatic residues of TAD2 to inhibit DNA binding.

A previous analysis of transcription factor-DNA complexes using the counterion condensation theory, notably HMG boxes and homeodomains, showed the salt-dependent component of binding was similar for specific and nonspecific DNA and the salt-independent components, attributed to hydrogen bonds and van der Waals interactions, were the drivers of specificity [50]. By contrast, our study shows that p53 has a larger salt-dependent component of binding for consensus DNA versus scrambled DNA; according to the counterion condensation theory, this represents a dependency on entropy derived from ion release when p53 binds consensus DNA that is not present when it binds the scrambled DNA sequence. Critiques of the counterion condensation theory have noted that ion release is not the only energetic component of the salt-dependent binding affinity, nor is the salt-dependent component entirely entropic [51,52,84,88]. Our data is discussed in the context of entropy derived from predicted ion release; however, our van’t Hoff data and previous ITC data [22] suggests a large enthalpic component, meaning the difference we see is a combination of ion release and other energetic components that drive specificity. 

Our results show how the presence of TAD2 decreases the apparent number of ions released by DBD when binding consensus DNA. We propose that the interactions between the positively charged residues in the DNA binding pocket and the negatively charged residues of TAD2 reduce the need for ionic interactions between those same positive charges of DBD and negatively charged solutes. This conclusion is consistent with the differences in ion release we see between the ND DE and ND NP mutants. The ND DE mutant releases almost the same number of ions as DBD. By eliminating the negative charges of TAD2 we have eliminated the intramolecular screening and now ions from the solute reestablish their positions around the positively charged amino acids of the DBD. The ND NP mutant has the negatively charged residues of TAD2 present, and the ion release is almost identical to that of ND WT. Thus, we show that the differences in ion release between DBD and ND WT are primarily moderated by negatively charged residues in TAD2. We also think the differences in the salt dependence of DNA binding between DBD and ND WT could be relevant for p53 function. Prior to DNA damage TAD1 is primarily responsible for the interaction with MDM2 that leads to p53 degradation [89]. However, following DNA damage, posttranslational modifications regulate numerous interactions between TAD2 and other cofactors [68,90,91,92]. It is reasonable to expect these other interactions will compete with the autoinhibitory function of TAD2, resulting in increased DNA binding.

## Figures and Tables

**Table 1:** ΔΔG (column-row) in kcal/mol at physiological IS.

Consensus DNA					
	DBD	ND DE	ND NP	ND QS	ND WT
**DBD**	0.00	1.03	1.13	1.49	3.02
**ND DE**	−1.03	0.00	0.10	0.46	1.99
**ND NP**	−1.13	−0.10	0.00	0.36	1.89
**ND QS**	−1.49.	−0.46	−0.36	0.00	1.53
**ND WT**	−3.02	−1.99	−1.89	−1.53	0.00
**Scrambled DNA**					
	**DBD**	**ND DE**	**ND NP**	**ND QS**	**ND WT**
**DBD**	0.00	0.46	0.64	0.63	0.97
**ND DE**	−0.46	0.00	0.18	0.17	0.51
**ND NP**	−0.64	−0.18	0.00	−0.01	0.32
**ND QS**	−0.63	−0.17	0.01	0.00	0.33
**ND WT**	−0.97	−0.51	−0.32	−0.33	0.00

**Table 2:** Slope of log (K_A_) versus log [Salt] predicts ion release.

	Slope, *N*, with Consensus DNA	Predicted Excess Ions released	Slope, *N*, with Scrambled DNA	Predicted Excess Ions released
**DBD**	−7.39	3.9	−4.09	0.6
**ND DE**	−7.08	3.6	−3.89	0.5
**ND NP**	−5.94	2.4	−6.91, −2.35	3.4, 0.0
**ND QS**	−7.16	3.6	−3.90	0.4
**ND WT**	−5.99	2.5	−4.15	0.7

**Table 3:** Stokes radii and apparent molecular weights of p53 constructs assessed by SEC.

	Stokes Radius (nm)	Elution Volume (mL)	Apparent Molecular Weight (kDa)	Actual Molecular Weight (kDa)
**DBD**	2.74 ± 0.004	63.78 ± 0.05	34.76 ± 0.13	24.55
**ND DE**	3.71 ± 0.001	52.41 ±0.01	67.89 ± 0.07	34.23
**ND NP**	3.65 ± 0.004	52.90 ± 0.04	65.98 ± 0.17	34.13
**ND QS**	3.65 ± 0.004	52.90 ± 0.02	65.98 ± 0.17	34.45
**ND WT**	3.55 ± 0.004	53.82 ± 0.04	62.46 ± 0.19	34.57

## Data Availability

Not applicable.

## Supplementary Materials

Supporting information can be downloaded at: https://www.mdpi.com/article/10.3390/biom12111558/s1: Table S1: K_D_s of p53 constructst binding consensus and scrambled DNA at various ionic strengths; Table S2: Standard error of estimate; Table S3: Percentage of Gibbs free energy originating from salt-dependent and salt-independent components estimated using Record’s interpretation of the counterion condensation model; Table S4: Percentage of Gibbs free energy originating from salt-dependent and salt-independent components estimated using Manning’s interpretation of the counterion condensation model; Table S5: Van’t Hoff plot-derived thermodynamics; Figure S1: Hill Coefficients of p53 constructs; Figure S2: ND WT binds DNA across ionic strengths; Figure S3: Salt-dependent and salt-independent components of Gibbs free energy at physiological ionic strength using Manning’s model; Figure S4: Van’t Hoff plots of DBD and ND with consensus and scrambled DNA; Figure S6: Separation of ND WT in seize exclusion column in low and high ionic strength buffer. References [76,93,94,95,96,97,98] are cited in Supplementary Materials.
