# Supplementary material for: Sequence Properties of an Intramolecular Interaction that Inhibits p53 DNA Binding
Source: Biomolecules. 2022 Oct 25;12(11):1558. doi: 10.3390/biom12111558 (PMC9687289; doi:10.3390/biom12111558)
Supplement: Supplementary file 1 [file biomolecules-12-01558-s001.zip › biomolecules-1962794-supplementary.pdf]

Supplementary materials for "Sequence dependence of an intramolecular interaction that inhibits DNA binding by p53"

Emily Gregory and Gary Daughdrill *

Department of Cell biology, Microbiology, and Molecular Biology,
University of South Florida, Tampa, FL 33620, USA
* Correspondence: gdaughdrill@usf.edu; Tel.: +1-813-974-2503

Supplementary Table S1: Kds (nM) of p53 constructs binding consensus and scrambled DNA at various ionic strengths.

	DBD		ND		ND DE		ND NP		ND QS	
IS	Con	Scram	Con	Scram	Con	Scram	Con	Scram	Con	Scram
**15mM**	0.018 ± 0.001	0.034 ± 0.004	0.71 ± 0.08	2.3 ± 0.1	NA	NA	NA	NA	NA	NA
**55mM**	0.61 ± 0.01	5.51 ± 0.03	0.97 ± 0.03	69 ± 3	NA	NA	NA	NA	NA	NA
**85mM**	2.08 ± 0.01	22 ± 2	7 ± 0.6	170 ± 10	2.1 ± 0.2	25.8 ± 0.2	1.4 ± 0.1	55 ± 6	3.08 ± 0.04	150 ± 6
**125mM**	0.90 ± 0.07	89 ± 5	43 ± 3	193± 8	4.6 ± 0.3	108 ± 2	3.2 ± 0.2	82 ± 2	7 ± 1	213 ± 9
**145mM**	1.39 ± 0.01	113 ± 7	230 ± 7	578 ± 40	7.6 ± 0.5	245 ± 7	9.4 ± 0.7	333 ± 7	17 ± 3	329 ± 25
**165mM**	7.1 ± 0.4	285 ± 25	473 ± 29	1500 ± 46.4	17.9 ± 0.7	593 ± 23	19 ± 3	830 ± 27	31.3 ± 0.7	855 ± 7
**185mM**	19 ± 2	590 ± 26	883 ± 49	1743 ± 105	96 ± 16	761 ± 69	52 ± 7	2006 ± 119	160 ± 9	1042 ± 58
**205mM**	58 ± 15	717 ± 62	1574 ± 163	2727 ± 66	290 ± 43	1083 ± 50	84 ± 6	2777 ± 85	347 ± 8	2402 ± 81
**225mM**	105 ± 5	1388 ± 44	3861 ± 40	3705 ± 230	384 ± 16	1676 ± 38	218.1 ± 15	3386 ± 31	889 ± 340	2760 ± 38

All values were obtained by fluorescence anisotropy. Each data point represents three titrations of protein into a stable concentration of DNA. ND mutants were not evaluated at ionic strengths below 85 mM, represented here as 'NA.'

Supplementary Table S2: Standard error of estimate.

	DBD		ND		ND DE		ND NP		ND QS	
	Con	Scram	Con	Scram	Con	Scram	Con	Scram	Con	Scram
**15mM**	0.0450	0.0744	0.0852	0.0003	NA	NA	NA	NA	NA	NA
**55mM**	0.0543	0.0180	0.0193	0.0198	NA	NA	NA	NA	NA	NA
**85mM**	0.0368	0.0161	0.0518	0.0134	0.0411	0.021	0.0186	0.0224	0.0351	0.0235
**125mM**	0.0310	0.0180	0.0254	0.0332	0.0276	0.0306	0.0402	0.032	0.0514	0.0274
**145mM**	0.0317	0.0173	0.059	0.0189	0.0332	0.0185	0.0345	0.0138	0.0377	0.0161
**165mM**	0.0318	0.0104	0.037	0.0323	0.0186	0.0168	0.0272	0.0121	0.0344	0.0438
**185mM**	0.0263	0.0243	0.0395	0.0201	0.0331	0.0379	0.0148	0.0173	0.0291	0.0178
**205mM**	0.0776	0.0249	0.0164	0.0172	0.0901	0.0248	0.0206	0. 0432	0.052	0.0087
**225mM**	0.0315	0.0250	0.0399	0.0212	0.082	0.0344	0.0433	0.0117	0.0368	0.0232

Triplicate runs of fluorescence anisotropy were evaluated using standard error of estimate as a measure of data quality using the following:

$$\frac{(Actual - Expected)^2}{dof}$$

Where *Actual* is the observed anisotropy values at a given concentration of protein and DNA, *Expected* is the predicted anisotropy value at these concentrations, and *dof* is degrees of freedom. ND mutants were not evaluated at ionic strengths below 85 mM, represented here as 'NA.'

Supplementary Table S3: Percentage of Gibbs free energy originating from salt-dependent and salt-independent components estimated using Record's interpretation of the counterion condensation model.

	Consensus DNA	
	% Electrostatic	% Nonelectrostatic
**DBD**	79.67	20.33
**ND DE**	82.34	17.66
**ND NP**	67.98	32.02
**ND QS**	85.05	14.95
**ND WT**	81.02	18.98
	**Scrambled DNA**	
	**% Electrostatic**	**% Nonelectrostatic**
**DBD**	55.95	44.05
**ND DE**	53.86	46.14
**ND NP**	97.68	2.32
**ND QS**	57.12	42.88
**ND WT**	60.56	39.44

Supplementary Table S4: Percentage of Gibbs free energy originating from salt-dependent and salt-independent components estimated using Manning's interpretation of the counterion condensation model.

	Consensus DNA	
	%Electrostatic	%Nonelectrostatic
**DBD**	68.97	31.03
**ND DE**	70.81	29.19
**ND NP**	46.56	53.44
**ND QS**	74.81	25.19
**ND WT**	63.00	37.00
	**Scrambled DNA**	
	**%Electrostatic**	**%Nonelectrostatic**
**DBD**	20.31	79.69
**ND DE**	19.39	80.61
**ND NP**	84.76	15.24
**ND QS**	18.00	82.00
**ND WT**	24.46	75.54

Supplementary Table S5: van't Hoff plot-derived thermodynamics. All values in kcal/mol.

Consensus DNA						
	**ΔH**	**ΔS**	**TΔS**	**ΔH-TΔS**	**Actual ΔG**	**Entropic %**
**DBD**	-5.252	0.022	6.601	-11.853	-11.838	56
**ND**	-2.843	0.021	6.198	-9.041	-9.047	69
**Scrambled DNA**						
	**ΔH**	**ΔS**	**TΔS**	**ΔH-TΔS**	**Actual ΔG**	**Entropic %**
**DBD**	-6.401	0.008	2.456	-8.856	-8.870	30
**ND**	-4.371	0.012	3.454	-7.825	-7.824	49

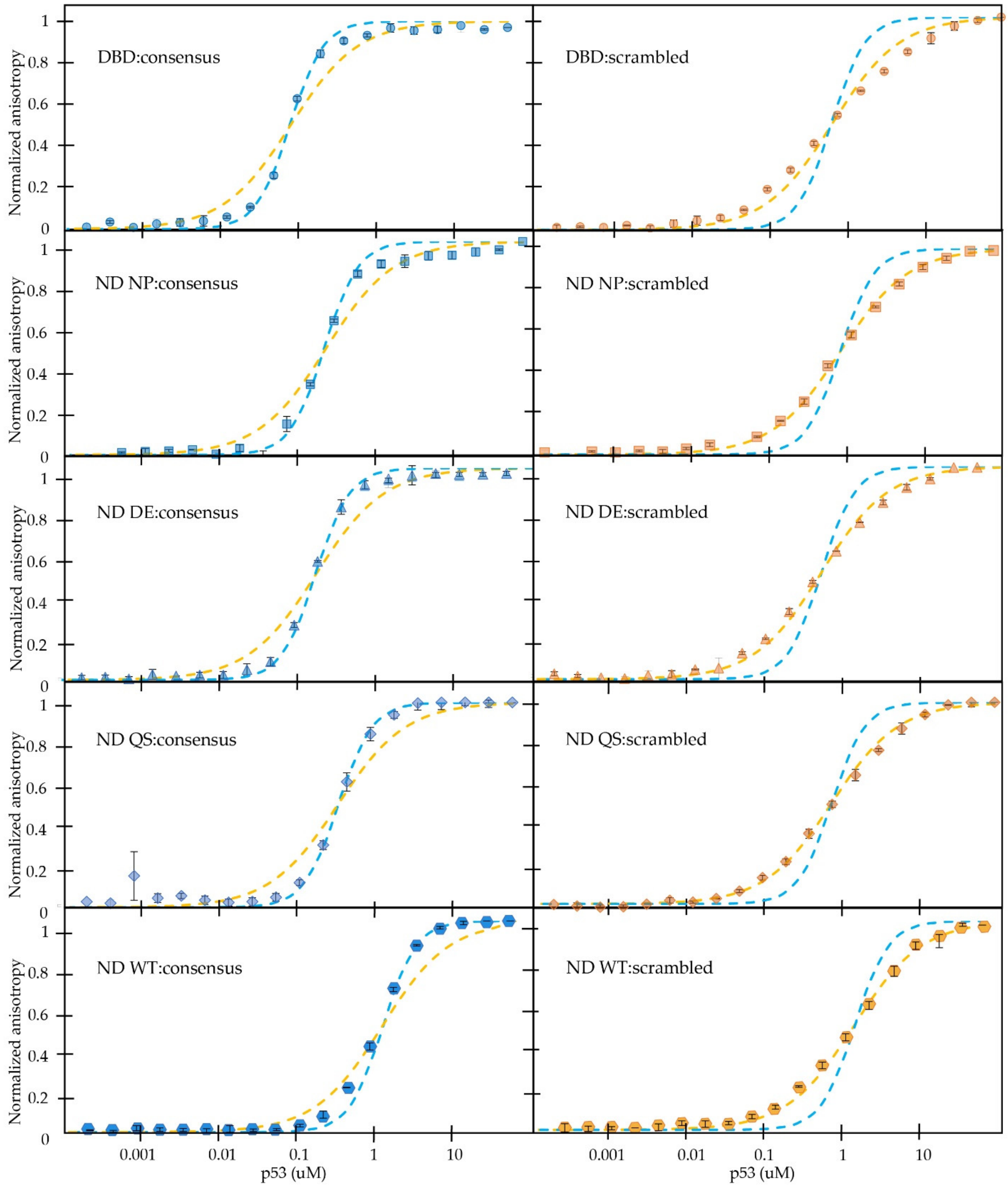

Supplementary Figure S1. Hill Coefficients of p53 constructs. Data points of p53 binding to consensus or scrambled DNA at 145mM ionic strength, filled shapes, are fit using an equation in which the Hill coefficient is set to 1 (orange dashed lines) or 2 (blue dashed lines) . Data for consensus DNA binding is best fit with a Hill coefficient of 2; data for scrambled DNA binding is best fit with a Hill coefficient of 1.

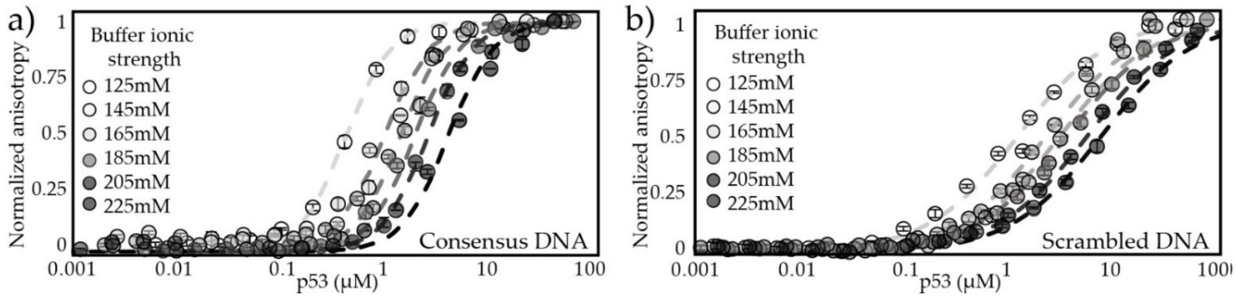

Supplementary Figure S2. ND WT binds DNA across ionic strengths. (a) fluorescence anisotropy plots of ND bound to consensus DNA at 125-225mM ionic strength; (b) fluorescence anisotropy plots of ND bound to scrambled DNA at 125 – 225mM ionic strength.

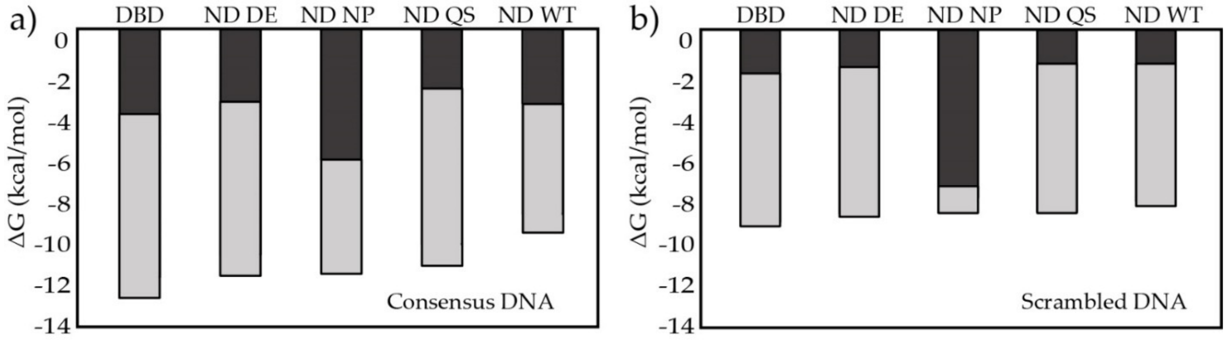

Supplementary Figure S3. Salt-dependent and salt-independent components of Gibbs free energy at physiological ionic strength using Manning's model where ■ is the salt-dependent component and □ is the salt-independent component for (a) consensus DNA, or (b) scrambled DNA.

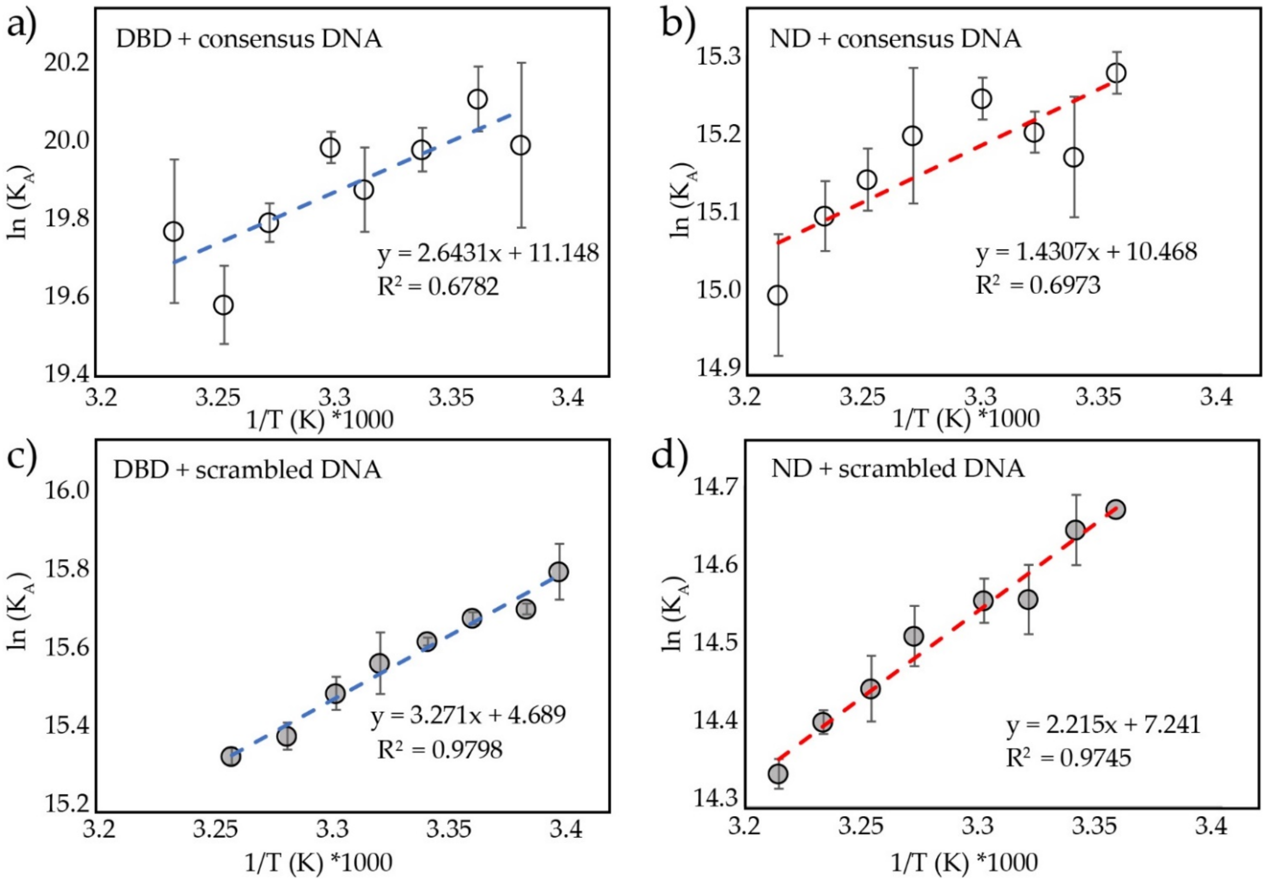

Supplementary Figure S4. Van't Hoff plots of DBD and ND with consensus and scrambled DNA derived from fluorescence anisotropy gives an estimate of enthalpic and entropic components of binding.

Thermodynamic values derived from a van't Hoff plot are inferred rather than directly measured. Enthalpic components such as buffer protonation and folding that occurs during binding is not measured here with the result that enthalpic values are always underestimated [1, 2]. The nearly linear plots of DBD and ND bound to scrambled DNA suggests minor heat capacity change [3], which supports predictions of DBD's structure when bound to nontarget DNA [4-6] and the general observation that nonspecific protein-DNA binding exhibits low heat capacity change [7]. The nonlinear plots of DBD and ND bound to consensus DNA suggest a non-negligible heat capacity change upon binding.

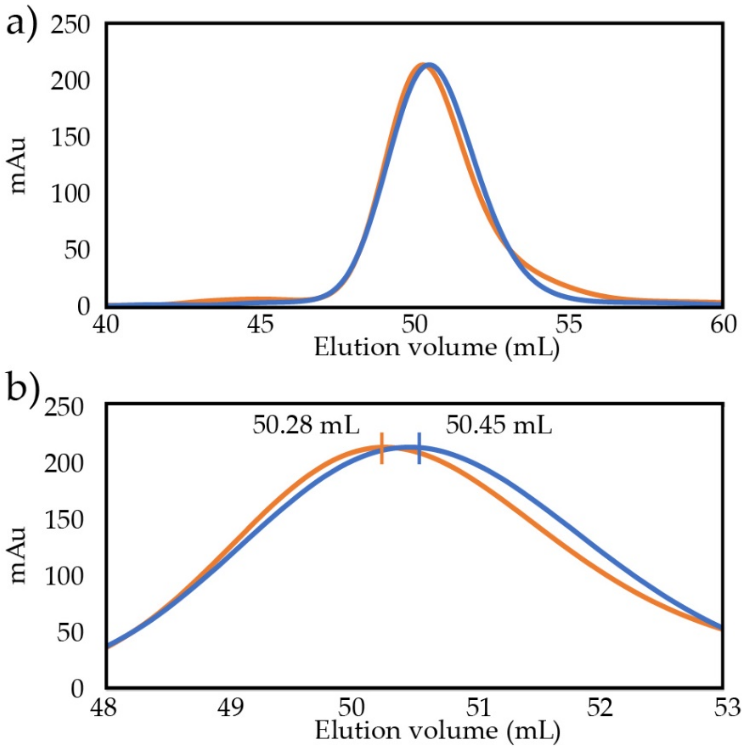

Supplementary Figure S5. Separation of ND WT in size exclusion column in low and high ionic strength buffer. a) ND WT in 150mM ionic strength buffer (orange line) and 410mM ionic strength buffer (blue line), b) inset of these with the maxima labeled. Note, elution volumes differ from that in main text Figure 7 because a different size exclusion column was used here; however, the Stokes radius of ND WT is the same as reported in the main text.

References

1. Liu, Y. and J.M. Sturtevant, *Significant discrepancies between van't Hoff and calorimetric enthalpies. II.* Protein Sci, 1995. **4**(12): p. 2559-61.
2. Datta, K., et al., *Temperature dependence and thermodynamics of Klenow polymerase binding to primed-template DNA.* Biophys J, 2006. **90**(5): p. 1739-51.
3. Zhukov, A. and R. Karlsson, *Statistical aspects of van't Hoff analysis: a simulation study.* J Mol Recognit, 2007. **20**(5): p. 379-85.
4. Demir, O., P.U. Ieong, and R.E. Amaro, *Full-length p53 tetramer bound to DNA and its quaternary dynamics.* Oncogene, 2017. **36**(10): p. 1451-1460.
5. Lambrughi, M., et al., *DNA-binding protects p53 from interactions with cofactors involved in transcription-independent functions.* Nucleic Acids Res, 2016. **44**(19): p. 9096-9109.
6. Melero, R., et al., *Electron microscopy studies on the quaternary structure of p53 reveal different binding modes for p53 tetramers in complex with DNA.* Proceedings of the National Academy of Sciences of the United States of America, 2011. **108**(2): p. 557-562.
7. Takeda, Y., P.D. Ross, and C.P. Mudd, *Thermodynamics of Cro protein-DNA interactions.* Proc Natl Acad Sci U S A, 1992. **89**(17): p. 8180-4.
